# Evaluation of the dynamic conformal arc therapy in comparison to intensity‐modulated radiation therapy in prostate, brain, head‐and‐neck and spine tumors

**DOI:** 10.1120/jacmp.v12i2.3197

**Published:** 2010-12-19

**Authors:** Manuel A. Morales‐Paliza, Charles W. Coffey, George X. Ding

**Affiliations:** ^1^ Department of Radiation Oncology Vanderbilt University Medical Center Nashville TN USA

**Keywords:** dynamic conformal arc therapy, intensity‐modulated radiation therapy, dose validation, treatment time, external‐beam radiation therapy

## Abstract

To evaluate dynamic conformal arc therapy (DAT) dose distribution and clinical applicability in comparison to intensity‐modulated radiotherapy (IMRT) in different types of tumors and locations, twelve patients with prostate cancer with no node involvement and three patients with single tumors in the pituitary, in the neck and in the thoracic spinal region treated with IMRT, were retrospectively planned with DAT using Eclipse (V8.1). The prostate cases were also planned with three‐dimensional conformal radiation therapy (3DCRT). Dose distributions were evaluated through comparisons of dose‐volumetric histograms and in‐house IMRT protocol constraints, as well as validated via ion chamber array measurements. DAT plans for prostate showed a statistically comparable achievement of tumor conformity and dose sparing for bladder and rectum when compared to IMRT. Dose on femoral heads were similar to those achieved using 3DCRT. DAT could be planned with similar results to those obtained in IMRT for the dose constraints of the defined structures by using a 360° arc for the brain lesion and several arcs including noncoplanar ones for the head‐and‐neck and spinal tumors. Experimental validation of the calculated dose distributions via gamma analysis of composite distributions for DAT provided that more than 95% of the pixels satisfy the criteria 3 mm–3%, which was similar to that of IMRT. The average number of monitor units was approximately five times lower than IMRT. In conclusion, DAT is capable of providing conformal dose distributions to the targets accomplishing many of the IMRT dose constraints simultaneously. Experimental dose‐validation accuracy, ease of planning and reduced treatment times make DAT both acceptable and attractive for clinical use.

PACS numbers: 87.55.D‐, 87.55.dk, 87.55.Qr, 87.56.bd, 87.56.Fc, 87.53.Kn, 87.55. de, 87.55.kd

## I. INTRODUCTION

The prime feature of intensity‐modulation radiation therapy (IMRT) is its ability to produce high‐dose gradients by virtue of dynamic or static use of a multileaf collimator (MLC) to deliver 2D fluence‐pattern intensities per beam which, in turn, were optimized by “inverse” planning. Due to this, IMRT is able to irradiate the prescribed dose to the tumor while sparing nearby organs at risk in a way that is usually unsurpassed by other techniques. The daily localization of the tumor and the adjacent surrounding^(^ ^1^ ^–^ ^3^ ^)^ is a challenge of treatment planning with high dose gradients. Both patient positioning and immobilization with IMRT are crucial to avoid dramatic underexposure to the target and/or overexposure to the nearby healthy tissue.

Furthermore, not only does IMRT planning and dose validation demand increased treatment efforts compared to other conventional external‐beam radiation therapy techniques, but also the dynamic/static MLC modulation in IMRT requires increased treatment times (monitor units). With the increased treatment times, there is an increased chance for dose‐delivery errors due to patient movement and/or internal organ motion during treatment. To reduce delivery error, image‐guidance techniques are often utilized (i.e., cone beam CT and respiratory gating),^(1)^ which further add extra complexity and treatment time. Another issue which may be associated with long periods of time to deliver a desired dose is biological effectiveness. Delivering the total dose in as short a time period as possible is thought to be more effective, since this will minimize the time available for repair of radiation‐induced DNA damage.^(^ ^4^ ^–^ ^5^ ^)^

For cases where static beams with no modulation are not sufficiently adequate to deliver dose to the tumor as well as sparing healthy organs, the additional option of rotating the gantry while shaping the MLC around the tumor may considerably improve the resulting dose distributions. This technique is known as dynamic conformal arc therapy (DAT). Additionally, the DAT technique will shorten treatment times and may reduce the need for complex image guidance procedures.

Alternatively, recent development and clinical application of intensity‐modulated arc therapy (IMAT) combines dynamic modulation of MLC with rotation of the gantry to deliver high dose gradient and very conformal dose distributions.^(6)^ The gantry rotation feature adds more optimization flexibility to improve conformity and deliver less number of monitor units than conventional IMRT dose distributions. However, the same IMRT target‐localization issues apply to IMAT as well.^(7)^

In this study, we present a few clinical cases of using DAT in comparison with IMRT. Dose distribution, tumor dose coverage, organs‐at‐risk dose sparing, as well as dose quality assurance are evaluated for DAT in comparison to the IMRT technique.

## II. MATERIALS AND METHODS

### A. Treatment planning system

The Eclipse external beam planning system version 8.1 including the AAA algorithm^(8)^ (Varian Medical Systems, Inc., Palo Alto, CA) for dose calculation was used to retrospectively plan cancer patients with DAT who had been recently treated using the IMRT technique. For each tumor, typical IMRT protocol specifications adopted in our clinic based on previous studies and public protocols were used as a benchmark in DAT planning. Additionally, for the prostate patients, three‐dimensional conformal radiation therapy (3DCRT) plans were developed. The same corresponding treated IMRT isocenters were used in all cases. The same contours for tumors and organs used in IMRT were utilized to perform the DAT (and 3DCRT) plans. The contours used for IMRT treatment had been defined according to our protocol specifications.

The planning strategy in all cases was to achieve the clinical IMRT constraints for the planning target volumes (PTV) or clinical target volumes (CTV) as much as possible, yet using forward planning on a trial‐and‐error basis. The DAT plans were forward‐planning optimized by varying the MLC margin per arc and the relative weight of the arcs. For the sake of an appropriate comparison, mean dose PTV/CTV matching with corresponding IMRT values for DAT plans (and 3DCRT plans, in the prostate case) was forced in this study. Dose volumetric histograms (DVHs) were obtained for each of these plans, from which parameters and IMRT protocol constraints of interest were extracted.

### B. Treatment sites

#### B.1 Prostate

Twelve patients with prostate cancer with no involvement of lymph nodes had been treated with IMRT using a typical 10 MV photon arrangement of coplanar beams (three patients with seven fields and nine patients with five fields). Each patient received a PTV dose of 76.00 Gy in 38 fractions. The prostate PTV had been delineated 0.8 cm around the prostate gross target volume (GTV), except in the posterior direction where a 0.6 cm margin was used to be reasonably away from the rectum. The seminal vesicles were contoured up to 1 cm above the prostate superiorly. The rectum contour was drawn between the anal verge and the sigmoid flexure.^(^ ^9^ ^–^ ^10^ ^)^ The bladder contour was defined including its cavity. The left and right femoral heads, as well as the urethra, were outlined in all cases. In four patients, small and/or large bowels were also defined. Table 1 shows the standard dose constraints used in the prostate IMRT protocol relevant for this study.

**Table 1:** Comparison of DAT and 3DCRT plans with IMRT for 12 patients with prostate cancer.

		*IMRT*	*DAT*			*3DCRT*	
*Organ [Prescription]*	*Parameter / IMRT Dose Limits (Gy)*	*Mean (STD)* ^a^ (%)	*Mean (STD)* ^a^ (%)	*p* ^b^ w/IMRT	*w/ 3DCRT*	*Mean (STD)* ^a^ (%)	*p* ^b^ w/ IMRT
Prostate PTV [76.00 Gy]	Min.=95%	*94.1* (4.0)	*94.8* (1.9)	0.6153	0.0001	97.9 (2.3)	0.0110
	Max.=105%	*105.4* (2.6)	104.3 (2.5)	0.0209	0.1618	103.8 (2.3)	0.0100
(Vol. / STD=156.5 / 56.3 cc)	DPTV‐95	99.9 (1.8)	99.0 (1.9)	0.0088	0.0001	100.2 (2.1)	0.1187
Prostate GTV	Min.=100%	100.2 (1.8)	100.0 (1.8)	0.4699	0.0647	100.5 (1.8)	0.0690
(Vol./STD=48.4/26.7 cc)	Max.=105%	104.2 (2.9)	103.9 (2.5)	0.3954	0.3251	103.6 (2.3)	0.1394
Bladder	DB−50<40(52.6%)	41.6 (15.1)	38.5 (29.0)	0.6707	0.0027	*57.9* (23.1)	0.0071
(Vol./STD=154.8/80.3 cc)	DB−20<65(85.5%)	75.3 (17.1)	78.0 (20.4)	0.2967	0.0047	*93.4* (10.6)	0.0005
	AUC‐B	47.2 (12.4)	43.3 (16.6)	0.2636	0.0001	61.1 (16.7)	0.0027
Rectum	DR‐60	26.6 (17.1)	32.1 (31.2)	0.4139	0.0101	55.2 (34.1)	0.0017
(Vol./STD=82.2/35.9 cc	DR−40<40(52.6%)	44.5 (23.1)	52.3 (36.7)	0.2361	0.0226	*71.9* (28.4)	0.0020
	DR‐30	57.6 (24.1)	65.8 (32.4)	0.1859	0.0157	83.6 (17.9)	0.0011
	DR−20<60(79.0%)	74.7 (23.0)	*84.1* (23.1)	0.0008	0.0658	*94.1* (9.9)	0.0022
	AUC‐R	41.6 (13.5)	46.5 (18.9)	0.0887	0.0029	61.1 (18.4)	0.0002
Left femoral head	DF−50<30(39.5%)	22.8 (11.3)	*48.0* (14.1)	<0.0001	0.8042	*48.7* (13.4)	<0.0001
(Vol./STD=131.6/66.4 cc)	DF−10<50(65.8%)	46.2 (9.2)	*66.2* (14.4)	0.0007	0.9303	*66.4* (18.4)	0.0033
	AUC‐F	26.5 (8.1)	46.3 (14.7)	<0.0001	0.3607	48.2 (13.8)	<0.0001
Urethra	${\text{DU}}_{\text{max}}=76(100\%)$	64.9 (35.4)	63.8 (38.5)	0.8046	0.0116	89.4 (16.0)	0.0157
Body $D_{\text{max}}=81.3(107\%)$	105.7 (2.5)	104.7 (3.1)	0.0969	0.0796	103.9 (2.3)	0.0069

a Mean values are given as percentages of prescribed dose (76 Gy). Mean dose values which do not meet the specific IMRT constraints are shown in italics.

b All p‐values which stand for statistical similarity (p≥0.0500) are underlined.

Abbreviations: STD=standard deviation; DXX‐YY is the dose at YY% of volume of XX structure; AUC‐YYY = area under the DVH curve for YYY structure normalized to ideal‐AUC‐PTV = (100% volume) (76 Gy); Vol. is the mean volume.

Following the Jereczek‐Fossa et al. study,^(11)^ two‐arc DAT coplanar beams (40°–140° and 220°–320°) and six‐field 3DCRT plans were developed for each patient. Photon beams with 10 MV of energy were used in all of these plans. The area‐under‐the‐curve (AUC) for some organs was defined as the area under the DVH for a specific structure, normalized to the ideal prostate‐PTV AUC (100% of volume times 76 Gy).^(^ ^12^ ^–^ ^15^ ^)^ In order to compare one technique with each other, paired Student's t‐tests were performed to assess the degree of similarity via the p‐value for some parameters between corresponding plans.^(16)^ A p=0.0500 value was selected as the threshold for statistical significance.

#### B.2 Brain tumor

A brain tumor (pituitary adenoma) treated with IMRT was selected to be planned with DAT. The GTV was fairly spherical, 5.3 cm^3^, and treated using two clinical tumor volumes (CTV1 and CTV2). The first one, CTV1, surrounded the GTV with an added margin of 0.5 cm and 54.00 Gy was prescribed to CTV1 in 30 fractions. The second one, CTV2, surrounded the GTV with an added margin of 1.5 cm and included the optic chiasm and part of the optical nerves. CTV2 was prescribed with 52.50 Gy to safeguard the optic chiasm and optical nerves for which the IMRT protocol allows maximum doses of 54 Gy. Table 2 shows the IMRT dose limits for the tumor and the critical structures. Due to the spherical nature of the tumor, one whole arc (0°–360°) was used to develop the DAT plan.

**Table 2:** IMRT and DAT doses for IMRT dose constraints for brain tumor and critical structures.

*Structure (Prescription)*	*IMRT Dose Limits (Gy)*		*IMRT Dose (Gy)*		*DAT Dose (Gy)*	
CTV1	Max.=105%	56.7	56.5		55.8	
(54.00 Gy)	Min.=95%	51.3	52.1		53.0	
CTV2	Max.=108%	56.7	*57.0*	0.001% V>56.7	55.8	
(52.50 Gy)	Min.=95%	49.9	*47.2*	0.6% V<49.9	*47.5*	0.6% V<49.9
Body	$D_{\text{max}}$	62	57.0		55.8	
Brain	D‐20	20	17.1		17.4	
	D‐10	30	26.3		28.9	
	D‐5	40	*42.1*	5.4% V>40	*47.7*	6.5% V>40
	$D_{\text{max}}$	45	*57.0*	4.6% V>45	*55.8*	5.5% V>45
Brainstem	$D_{\text{max}}$	54	53.8		*54.1*	0.3% V>54
Left Eye	D‐20	35	16.2		21.3	
	D‐10	45	19.7		22.2	
Left Lacrimal	D‐50	20	7.2		13.5	
Left Optic Nerve	$D_{\text{max}}$	54	46.7		53.8	
Optic Chiasm	$D_{\text{max}}$	54	52.9		*54.8*	26.4% V>54
Right Eye	D‐20	35	19.6		17.3	
	D‐10	45	21.8		17.8	
Right Lacrimal	D‐50	20	*21.1*	64.1% V>20	16.2	
Right Optic Nerve	$D_{\text{max}}$	54	52.8		*55.4*	29.6% V>54
Skin	D‐20	20	5.2		5.7	
	D‐10	30	8.7		10.3	
	D‐5	40	12.2		12.4	
	$D_{\text{max}}$	50	37.1		26.7	

Abbreviations: D‐YY is the dose at YY% of volume; V is volume; doses which do not satisfy IMRT dose limits are shown in italics; the corresponding above/below‐dose volumes follow.

#### B.3 Neck tumor

A parapharyngeal‐paraspinal chondroma in the neck that had been treated with IMRT was planned with DAT. The clinical tumor volume CTV1 was defined having a volume of 97.9 cm^3^ and was prescribed with 65.92 Gy in 32 fractions. A margin of 1 cm was added around it to define CTV2 which was prescribed with 54.40 Gy. CTV2 included portions of the oral cavity, right parotid and larynx. Table 3 contains the IMRT constraints for the targets and organs at risk. Because of the large size and irregular shape of the tumor, as well as its relative position within the neck, four arcs, including two noncoplanar arcs, were used for the plan. The arc that irradiates the more superficial extent of the tumor was planned using 6 MV as the photon energy, whereas the other three arcs were planned with 18 MV photons.

**Table 3:** IMRT and DAT doses for IMRT dose constraints for nasopharyngeal tumor and critical structures.

*Structure (Prescription)*	*IMRT Dose Limits (Gy)*		*IMRT Dose (Gy)*			*DAT Dose (Gy)*
CTV1	Max.=108%	71.2	70.9		69.8	
(65.92 Gy)	Min.=95%	62.6	*61.5*	0.01% V<62.6	*58.1*	0.3% V<62.6
CTV2	Max.=108%	58.8	*70.9*	70.7% V>58.8	*69.8*	91.1% V>58.8
(54.40 Gy)	Min.=95%	51.7	*42.4*	0.8% V<51.7	*33.5*	2.1% V<51.7
Back	D‐50	20	15.5		0.3	
	D‐20	30	29.4		16.3	
	D‐10	40	35.8		30.1	
	$D_{\text{max}}$	50	*70.4*	1.7% V>50	*67.3*	3.1% V>50
Body	$D_{\text{max}}$	72	70.9		69.8	
Brain	D‐20	20	0.9		0.5	
	D‐10	30	1.6		0.9	
	D‐5	40	4.2		1.9	
	$D_{\text{max}}$	45	4.4		24.6	
Larynx	D‐50	20	19.8		*27.6*	59.3% V>20
	D‐20	30	*36.6*	28.7% V>30	*57.8*	47.5% V>30
	D‐10	40	*47.1*	16.6% V>40	*62.9*	38.6% V>40
	$D_{\text{max}}$	50	*62.6*	7.0% V>50	*66.9*	29.6% V>50
Left Parotid	D‐50	20	8.9		4.6	
Oral Cavity	D‐50	20	*20.9*	52.5% V>20	*46.0*	80.0% V>20
	D‐20	30	*31.7*	22.8% V>30	*61.1*	68.3% V>30
	D‐10	40	*44.6*	12.4% V>40	*64.0*	57.1% V>40
	$D_{\text{max}}$	50	*66.6*	7.2% V>50	*67.7*	44.3% V>50
Right Parotid	D‐50	20	*31.0*	77.9% V>20	*50.7*	84.4% V>20
Skin	D‐20	20	2.2		5.0	
	D‐10	30	8.7		11.5	
	D‐5	40	15.4		19.1	
	$D_{\text{max}}$	50	*63.4*	0.4% V>50	*60.1*	0.2% V>50
Cervical Cord	$D_{\text{max}}$	45	*49.2*	0.1% V>45	*47.8*	0.04% V>45

Abbreviations: D‐YY is the dose at YY% of volume; V is volume; doses which do not satisfy IMRT dose limits are shown in italics; the corresponding above/below dose volumes follow.

#### B.4 T‐spine tumor

A leiomyosarcoma in the T2‐T4 thoracic spine was treated with a seven‐field IMRT plan which included two noncoplanar fields. A corresponding DAT plan was developed retrospectively for the same tumor. The clinical target volume CTV was contoured generating a 198.8 cm^3^ target volume to which a 57.20 Gy dose delivered in 26 fractions was prescribed. A 1 cm margin was added to CTV to define the PTV as a control structure for IMRT optimization purposes. The IMRT dose limits and constraints for the tumor and other critical organs are shown in Table 4. Two 18 MV photon coplanar arcs in addition to one 6 MV photon noncoplanar arc were used to obtain the best possible results with this large irregularly shaped tumor.

**Table 4:** IMRT and DAT doses for IMRT dose constraints for spine tumor and critical structures.

*Structure (Prescription)*	*IMRT Dose Limits (Gy)*		*IMRT Dose (Gy)*		*DAT Dose (Gy)*	
CTV	Max.=105%	60.1	*62.9*	9.2% V>60.1	59.6	
(57.20 Gy)	Min.=95%	54.3	*51.4*	0.03% V<54.3<	*44.1*	1.6% V<54.3<
PTV	Max.=105%	60.1	*62.9*	6.5% V>60.1	59.6	
(57.20 Gy)	Min.=95%	54.3	*30.6*	4.2% V<54.3<	*26.9*	23.8% V<54.3<
Body	$D_{\text{max}}$	62.9	*62.9*	0.001% V>62.9	59.6	
Esophagus	D‐100	45	0.01		0.01	
	D‐66	55	3.5		0.8	
	D‐33	60	13.9		10.2	
Heart	D‐100	30	0.01		0.01	
	D‐66	45	1.0		0.2	
	D‐33	60	2.3		0.4	
Left Lung	D‐50	15	2.8		1.4	
	D‐35	20	4.6		4.9	
Right Lung	D‐50	15	10.7		3.0	
	D‐35	20	15.4		11.3	
Both Lungs	D‐50	15	6.0		2.3	
	D‐35	20	10.1		6.4	
Spinal Cord	$D_{\text{max}}$	45	36.8		39.3	
Skin	D‐20	20	6.4		7.3	
	D‐10	30	12.7		11.9	
	D‐5	40	19.6		17.4	
	$D_{\text{max}}$	50	*62.9*	1.7% V>50	*57.8*	0.9% V>50

Abbreviations: D‐YY is the dose at YY% of volume; V is volume; doses which do not satisfy IMRT dose limits are shown in italics; the corresponding above/below‐dose volumes follow.

### C. Dose validation system

The ion chamber array system MatriXX embedded into the MultiCube phantom (IBA Dosimetry, Germany) was used to validate integrated (composite) fields for both IMRT and DAT plans in one prostate and in the brain tumor cases. The phantom was imaged by computed tomography and acquired into our treatment planning system. Each IMRT and DAT plan were mapped on this phantom with the same field arrangements and monitor units, matching the frontal isocenter plane with the dosimeter array plane. Combined dose distributions were calculated using the same treatment planning system algorithm. Then, these plans were delivered on the phantom using a linear accelerator, and combined absorbed dose distributions were measured. The measured absorbed dose distribution for the frontal isocenter plane, filtered using a cubic‐spline smoothing function, was compared to the calculated one for each plan using the OmniPro‐I'mRT software platform (IBA Dosimetry, Germany) by determining the percentage of points (pixels) with a gamma factor (3 mm distance‐to‐agreement and 3% dose difference) greater than one in the entire detector plane (24 cm×24 cm) with 1.25 mm of grid size.^(17)^

## III. RESULTS

**Figure 1:**
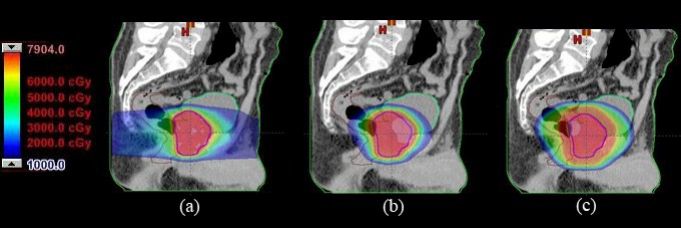
Isodose distribution at the sagittal isocenter plane for IMRT (a), DAT (b) and six‐field 3DCRT (c).

**Figure 2:**
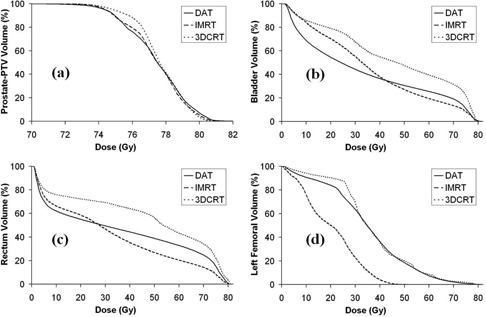
Mean dose‐volumetric histograms for prostate PTV (a), bladder (b), rectum (c) and left femoral head (d), corresponding to 12 patients with prostate cancer.

Figure 1 shows the sagittal dose distribution for one typical prostate cancer case, while Fig. 2 depicts the mean DVHs for prostate‐PTV, bladder, rectum and left femoral head for the three different techniques. Mean values of dose, AUC and volume, as well as comparison between the techniques analyzed with t‐tests, are presented in Table 1 for organs of interest for each individual technique.

The mean minimum and maximum dose values for prostate‐PTV are better achieved with 3DCRT. Likewise, Fig. 2(a) and the doses received by 95% of the prostate‐PTV volume (DPTV‐95) for the three different techniques reveal that 3DCRT presents the highest volume/dose slope for prostate‐PTV, although all the three maximum slopes are very close. To verify this, the heterogeneity index (HI)^(18)^ was calculated for the mean prostate‐PTV DVHs, providing HI values of 1.03, 1.06, and 1.08 for 3DCRT, DAT and IMRT, respectively. One possible explanation is that the six static symmetrically arranged coplanar beams provide a slightly better chance to get a higher degree of uniformity in dose distribution than the more lateral arc coverage in DAT and, in a lesser degree, the additional constraints imposed for the other organs in inverse IMRT planning. The seminal vesicles received a statistically similar range of doses (not shown) for DAT and 3DCRT plans compared with IMRT (p‐values greater than 0.0500 for mean minimum and maximum doses). Thus, all the targets received a comparable dose distribution from each of the three treatment techniques, allowing organs at risk dose comparison for each technique.

The mean doses received at 50% and 20% of bladder volume (DB‐50 and DB‐20) and those received at 60%, 40% and 30% of rectum volume (DR‐60, DR‐40 and DR‐30), as well as the mean AUCs for these two organs, show through the t‐tests that DAT is statistically comparable to IMRT in bladder and rectum doses (p‐values > 0.0500). On the other hand, the 3DCRT exhibits mean doses and AUC for bladder and rectum that are not statistically similar to those of IMRT (p‐values < 0.0500). Only the DAT mean dose at 20% of rectum volume (DR‐20) is statistically different than IMRT (p=0.0008) but similar to 3DCRT (p=0.0658). These results strongly suggest that DAT is statistically comparable to IMRT in terms of bladder and rectum dose sparing while providing the same mean dose to the prostate PTV. On the other hand, 3DCRT is much less effective in sparing these two organs than DAT and IMRT, under the same target conditions.

Mean doses at 50% and 10% of left femoral head volumes (DF‐50 and DF‐10), the mean‐femoral AUC (AUC‐F) and the DVHs in Fig. 2(d) clearly show that IMRT is the only technique that spares the femoral heads in such a way that the imposed IMRT dose constraints to these organs are satisfied. In fact, DAT and 3DCRT are statistically similar (p‐values > 0.0500) in terms of these dose indicators. The right femoral head (not shown here) provided similar results as the left femoral head.

Maximum doses for urethra and body are presented in Table 1. The maximum dose values for urethra are very similar for DAT and IMRT (p=0.8046), and these maximum values are much less than the ones for 3DCRT. This indicates that even though no specific dose constraint was imposed to the urethra in the DAT plans, the lateral arcs act in such a way that the DAT maximum dose satisfies this IMRT constraint. The maximum dose in the body was always outside the organs at risk in all the plans; the mean value for all the techniques is less than 107%, exhibiting the highest mean value in the IMRT case.

In general, mean dose values with IMRT protocol constraints in Table 1 reveal that for bladder and urethra the IMRT protocol constraints can be fully reached with DAT. In the case of rectum, the IMRT constraint for DR‐40 is achievable with DAT, while for DR‐20 it is not. The 3DCRT plans, on the other hand, were not able to spare the rectum and bladder organs to obtain the dose limits imposed by the IMRT protocol constraints. Both DAT and 3DCRT could not achieve the IMRT constraints for the femoral heads.

The mean numbers of monitor units (MU) needed to deliver these prostate plans were as follows: 966 with standard deviation (STD) of 235 MU for IMRT, 319 with STD of 35 MU for DAT, and 287 with STD 28 MU for 3DCRT. Therefore, considering, in addition, the 5–7 beams in IMRT, 6 beams in 3DCRT, and the two arcs in DAT, the dose‐delivery times for treatment are considerably reduced by using DAT than IMRT or 3DCRT.

**Figure 3:**
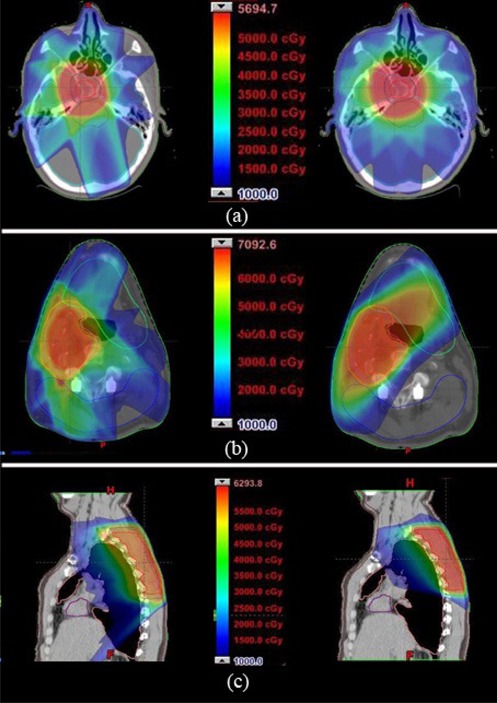
Isodose distribution at the isocenter plane for IMRT (left panel) and DAT (right panel) for the brain (axial) (a), head‐and‐neck (axial) (b) and spine (sagittal) (c) lesions.

**Figure 4:**
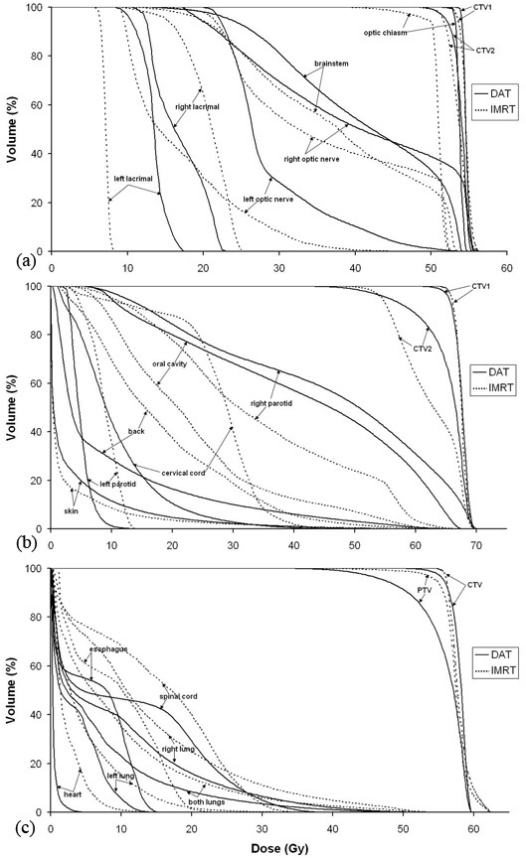
Dose‐volumetric histograms of tumors and organs at risk for brain (a), head‐and‐neck (b), and spine (c) cases.

Table 2 compares the obtained dose values for the pituitary‐brain tumor treated with IMRT and planned with DAT. (Figures 3a)and (4a) show the corresponding isodose distributions and the DVHs, respectively, for IMRT and DAT. The conformity of the CTV1 and CTV2 coverage as well as the right‐lacrimal gland and right eye sparing are better for DAT. However, while the high IMRT dose gradient allowed for optic chiasm, optical nerves and brainstem dose maximums below the limit (54 Gy), the 360° arc DAT plan delivered higher maximum doses for the optic chiasm and the right‐optic nerve, as shown in Table 2. Likewise, the accumulated number of monitor units for the five‐field IMRT plan is 798 MU, while the one‐arc DAT plan takes only 234 MU.

Table 3 provides values for doses associated to the protocol IMRT dose constraints for both the treated IMRT and the DAT plans for the neck lesion. Likewise, (Figs. 3b)and (4b) show the corresponding isodose distributions and DVHs for both techniques. Conformity for CTV1 is slightly better for IMRT. CTV2 dose distribution deviates less from their limits for IMRT, as well. However, cervical‐cord sparing is much better for DAT. Except for the oral cavity and right parotid, all the other organs at risk for DAT are better or comparable to IMRT in terms of dose sparing. The five‐field IMRT plan accumulated 1360 MUs, while the four‐arc DAT plan presents a cumulative number of 225 MUs.

Table 4 and (Figs. 3c)and (4c) show that the spine tumor coverage in CTV is slightly better for IMRT, yet very acceptable for DAT; however, the dose sparing of almost all the organs at risk is better achieved by DAT. Also, the global hot spot is lower for DAT. The total number of MUs for the seven‐field IMRT is 1736, while the total MUs for the three‐arc DAT plan is only 265.

**Figure 5:**
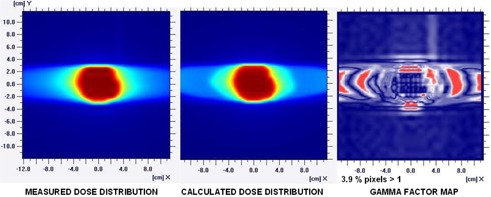
Measured and calculated dose distribution profiles of the frontal isocenter plane with gamma analysis (3 mm and 3%) for the brain tumor DAT plan.

Experimental validation was carried out by measuring combined absorbed dose profiles, which were compared to the calculated ones for composite fields/arcs for both IMRT and DAT plans in one prostate and in the brain tumor cases. The absolute dose was within 2% in the high‐dose/low gradient portion of the distribution for both DAT and IMRT plans, with no significant difference. The percentage of pixels with a gamma factor (3 mm and 3%) higher than 1 was lower than 5% in all cases, indicating that use of this two‐dimensional ion chamber array provides an acceptable way of assessing dose‐distribution comparisons. Particularly, the percentage of pixels with a gamma factor higher than 1 was 3.9% for the DAT brain case, depicted in Fig. 5.

## IV. DISCUSSION

This study shows that targets at different sites can be adequately treated with DAT, providing a cost‐effective advantage in treatment time when compared to IMRT, yet keeping a similar dose uniformity of the target and an acceptable dose tolerance to the organs at risk. We showed that, by choosing appropriate arcs based on tumor shape and relative location to organs at risk, approximation to IMRT dosimetric results is feasible. Ultimately, selection of a DAT plan versus an IMRT one should be based on some flexibility regarding IMRT constraints which, in turn, depends on each specific patient.

IMRT has become the regular choice for radiation therapy of prostate cancer since modulation reduces the rectum dose considerably. However, in cases where the lymph nodes do not need to be treated, our results show that our protocol IMRT constraints can be reasonably achieved for bladder and rectum by applying two 100° wide arc DAT. Femoral heads in DAT are, however, not as well spared as in IMRT, yet they are not worse than 3DCRT. Jereczek‐Fossa et al.^(11)^ obtained mean values for DPTV‐95, DB‐50, AUC‐B, DR‐60, DR‐30, AUC‐R, DF‐50, and AUC‐F for DAT and 3DCRT. The results presented here closely matched the earlier findings of this study. In a recent clinical study involving treatment of 542 patients with prostate cancer using two 100° wide arc DAT, Jereczek‐Fossa et al.^(19)^ demonstrated that dose escalation from 76 Gy/38 fractions to 80 Gy/40 fractions does not increase toxicity; unfortunately, their results have not produced better tumor outcome. From the bladder DVHs in Fig. 2(b), below 40 Gy (“low dose”), DAT seems to be the best technique in sparing low doses in the bladder, while above 40 Gy (“high dose”), IMRT produces the least high‐dose toxicity. At full prescribed dose, the mean volume percentages of bladder receiving 76 Gy are 7.0%, 8.4% and 17.3% for IMRT, DAT and 3DCRT, respectively. As a whole, the mean AUC‐B indicates a slightly lesser number for DAT than for IMRT, although statistically both techniques should be considered similar (p=0.2636). (Figure 2c) shows that for rectum the threshold for “low” and “high” doses to distinguish between higher DAT or IMRT toxicity is lower than that of bladder; yet, mean AUC‐R values are statistically similar between DAT and IMRT (p=0.0887). At the higher dose range, the mean volume percentages of rectum receiving 76 Gy provide values of 6.5%, 11.6% and 17.8% for IMRT, DAT and 3DCRT, respectively. How a lower or a higher dose‐volume distribution of toxicity in the bladder or rectum using this radiation‐fractionation scheme for prostate cancer ultimately affects these organs is still a topic for further study.^(^ ^19^ ^–^ ^20^ ^)^ Sasaoka et al.^(21)^ showed that a combination of five‐field 3DCRT and DAT can reduce the rectal dose compared to a five‐field 3DCRT or DAT only. Shiraishi et al.^(22)^ showed that two dynamic‐arc therapies with half rotation around two isocenters provided equivalent sparing of normal structures to IMRT, although with inferior target‐dose uniformity. Alternatively, Metwaly et al.^(23)^ have demonstrated that a combination of dynamic arcs and two lateral‐oblique conformal fields may produce further protection to the rectum. All these studies, including this investigation, reveal the flexibility of DAT forward planning in low‐risk prostate cancer as well as its suitability for dose escalation.

The DAT plan for the pituitary‐brain tumor satisfies most of the corresponding IMRT constraints, as presented in Table 2. By choosing different arcs and appropriate angles, the dose exposure on the eyes and lacrimal glands can be reduced considerably, if required. We developed two‐arc coplanar and four noncoplanar arc plans, which deliver a dose reduction in these organs but at the expense of a dose increase in the optic chiasm, optic nerves and brainstem. Thus, if the priority is to protect these latter organs, the one‐360° arc plan is the best option.

The fact that dose sparing for the cervical cord in the head‐and‐neck case while keeping a comparable tumor coverage is much better with DAT makes the DAT plan an attractive alternative to IMRT. The modulation power of IMRT allows for high dose gradients between CTV1 and CTV2, but by optimizing under this criterion, some organs at risk (such as the cervical cord, in this case) do not get much dose sparing. However, in this particular plan, the oral cavity and the right parotid received a considerable greater amount of dose using DAT, which would need to be evaluated and considered against the cervical‐cord advantages.

By simple inspection of the IMRT and DAT plans for the spine case, it can be concluded that DAT is dosimetrically comparable – or even better – than IMRT for the sparing of most of the organs, keeping a very close CTV coverage. The sagittal views of Fig. 3(c) reveal a much less invasive dose in the right lung with DAT compared to IMRT. Bral et al.^(24)^ have performed a clinical study of an image‐guided hypofractionated DAT for inoperable patients with lung cancer, concluding that application of DAT in this type of cancer is feasible. Interestingly, Piermattei et al.^(25)^ have developed a novel method for the determination of the *in vivo* isocenter dose using a small ion chamber and electronic portal imaging, which can be applied to DAT in thoracic tumors.

This study also evaluated the variation of the tumor dose coverage by moving the isocenter 2 mm away from the one in the original plan. Comparing the shifted and non‐shifted tumor DVHs, DAT dose shows less sensitivity than the corresponding IMRT dose. This result was expected, given the higher dose gradients in IMRT plans, which depends heavily on steep dose gradients between the tumor and organs at risk.^(26)^ Thus, DAT plans offer less chance for deviation from the prescribed dose due to setup errors and/or internal motion of the organs than IMRT.

Given that the number of monitor units for DAT is considerably smaller than in IMRT for all the plans presented in this study, the treatment delivery is fast. By the same token, chances of internal organ motion during delivery are reduced, and achievability of on‐board imaging can be, therefore, more surely assessed. The MU factors between IMRT and DAT plans in this study were 3.03 for prostate (average), 3.41 for brain, 6.04 for head and neck, and 6.55 for spine. Considering that the dosimetry of these plans may be clinically acceptable, the significant reduction in the number of monitor units for DAT make these plans attractive as an alternative to IMRT, for both the comfort of the patients during treatment and the associated reduced costs in treatment time.

## V. CONCLUSIONS

We have shown that DAT has the potential to be an alternative to IMRT and conventional planning for tumors where there is flexibility in dose constraints to organs at risk. DAT plans with satisfactory dose limitations were developed for prostate, brain, head‐and‐neck and spine tumors. To validate the dose distribution and absolute values in these plans, DAT quality assurance was performed and validated by percentages of pixels with a gamma factor higher than 1, presenting values less than 5%. This study indicates that implementation of DAT to treat patients with prostate cancer with no lymph node implication, as well as for brain, head‐and‐neck and spine tumors, is clinically viable.

## ACKNOWLEDGMENTS

The authors would like to acknowledge the help of Mr. Horace Lambert who was an AAPM‐minority summer student at our institution. This work was conducted in part using the resources of the Varian research grant 31395‐R.
